# Non-ischemic laparoscopic partial nephrectomy using 1318-nm diode laser for small exophytic renal tumors

**DOI:** 10.1186/s12894-018-0405-9

**Published:** 2018-11-09

**Authors:** Martin Drerup, Ahmed Magdy, Martina Hager, Daniela Colleselli, Thomas Kunit, Lukas Lusuardi, Günter Janetschek, Michael Mitterberger

**Affiliations:** 1 Department of Urology, Paracelsus Medical University Salzburg, Muellner-Hauptstrasse 48, 5020 Salzburg, Austria; 2 Department of Pathology, Paracelsus Medical University Salzburg, Salzburg, Austria

**Keywords:** Renal cell carcinoma, Non ischaemic nephrectomy, Laparoscopy, Laser

## Abstract

**Purpose:**

Warm ischemia (WI) and bleeding constitute the main challenges for surgeons during laparoscopic partial nephrectomy (LPN). Current literature on the use of lasers for cutting and coagulation remains scarce and with small cohorts. We present the largest case series to date of non-ischemic LPN using a diode laser for small exophytic renal tumors.

**Methods:**

We retrospectively evaluated 29 patients with clinically localized exophytic renal tumors who underwent non-ischemic laser–assisted LPN with a 1318-nm wavelength diode laser. We started applying the laser 5 mm beyond the visible tumor margin, 5 mm away from the tissue in a non-contact fashion for coagulation and in direct contact with the parenchymal tissue for cutting.

**Results:**

The renal vessels were not clamped, resulting in a WIT (warm ischaemic time) of 0 min, except for one case that required warm ischemia for 12 min and parenchymal sutures. No transfusion was needed, with a mean Hemoglobin drop of 1,4 mg/dl and no postoperative complications. The eGFR did not significantly change by 6 months. Histologically, the majority of lesions (*n* = 22/29) were renal-cell carcinoma stage pT1a. The majority of malignant lesions (*n* = 13/22) had a negative margin. However, margin interpretation was difficult in 9 cases due to charring of the tumor base. A mean follow-up of 1.8 years revealed no tumor recurrence. The mean tumor diameter was 19.4 mm.

**Conclusion:**

The 1318-nm diode laser has the advantages of excellent cutting and sealing properties when applied to small vessels in the renal parenchyma, reducing the need for parenchymal sutures. However, excessive smoke, charring of the surgical margin, and inability to seal large blood vessels are encountered with this technique.

## Introduction

Renal cell carcinoma is one of the more frequent carcinomas, comprising 2–3% of all cancers. Within the last few years, the incidence has consistently increased in most Western countries. [1] A shift to more organ-confined tumors has been observed due to diagnostic changes, such as ultrasound and computer tomography. [2] The only feasible therapy to date is surgical extirpation. The surveillance for patients with SRM is under investigation. Currently, it is feasible strategy both for hereditary RCC patients and those with small renal masses with more indolent growth rates. Recent guidelines point out that NSS should be performed in all tumors < 7 cm whenever feasible. There are no differences in the overall survival and cancer-specific survival between radical nephrectomy and partial nephrectomy (PN), with the advantage of better renal function preservation in PN patients. [3] Therefore, it is a goal to perform nephron-sparing surgery whenever feasible by sparing the maximum amount of renal parenchyma and reducing warm ischemic time (WIT). In order to achieve a good surgical result with restricted blood loss, hilar clamping is the standard procedure. The interruption of renal blood flow leads to WIT, with a loss of renal function. [4] Patients who already suffer a chronic renal insufficiency have the highest risk to loose further renal function due to WIT. In these patients, hypoxic radicals formed in the hypoxic tissue after off clamping can lead to further damage within 5–8 min. [5] The most common complication in NSS remains bleeding, with a transfusion risk of 5%. [6] To optimize the surgical treatment of RCC, the technique is under continuous investigation. Improvements have been done over the years in reducing the risk of bleeding, as well as reducing WIT and complications of any cause. By changing the access from open to laparoscopic partial nephrectomy (LAPN), and more recently to robot-assisted partial nephrectomy (RAPN) the procedure was upgraded. [7] With laparoscopic access, the intraoperative blood loss is lower than with the open surgical approach, whereas the postoperative complications do not increase. [8] However, LAPN elevates the WIT, as the surgical steps in a crucial time frame are challenging, even for an experienced surgeon. [9] Therefore, different surgical techniques have emerged to reduce or eliminate the WIT, including targeted renal blood flow interruption, selective renal artery clamping, selective renal parenchymal clamping, laser supported MIPN, radio frequency assisted MIPN, hydro-jet assisted MIPN, and sequential preplaced suture renorrhaphy. [10] None of these techniques are widely accepted, and they are still under investigation. Thus, we chose to further investigate the laser for partial nephrectomy in this case-control study. Different wavelengths have different absorptions in different types of tissue, and finding the optimal wavelength is crucial. Experience in non-ischemic laparoscopic partial nephrectomy has been gained with a Ho:YAG-, Thulium-,and Diode-laser. Ho:YAG, ﻿Nd:YAG and KTP have good cutting and coagulation properties with the drawback of blood splashing and excessive smoke building due to the pulsating nature of the Ho:YAG laser. Thulium and Diode laser are the most recent investigated promising laser systems with excellent cutting and coagulation features. Smoke building and carbonization of the tissue is also observed [11].

As different types of lasers have already been tested, the aim of this study was to show the feasibility of laser laparoscopic partial nephrectomy (LLPN) using a 1318-nm diode laser. Tumor excision and the ability of pathological reporting after laser enucleation was also of interest when the oncological result coincided with the actual standard of care.

## Materials and methods

Between May 2012 and June 2014, we retrospectively evaluated 29 patients with clinically localized exophytic renal tumors. All patients were operated on at the university clinic Salzburg using a 1318-nm laser. The ethics committee was consulted. The diagnosis of SRM and decision for surgery with laparoscopic laser enucleation was based on CT scans and/or magnetic resonance tomography. The patient who underwent surgery had mainly exophytic renal masses with a minimum distance of 5 mm to the renal calix. Renal masses were included in the study when radiologically suspected of malignancy. All patients were classified by PADUA score and R.E.N.A.L score [12] for an objective preoperative evaluation of the complexity of the intervention. Only single lesions were treated in this study, and only renal masses up to 4 cm in maximum dimension. Centrally located tumors were excluded, as well as patients with a single functional kidney.

### Instruments and laser

The renal masses were enucleated by non-ischemic LLPN using the Eraser® laser (Rolle & Rolle, Salzburg, Austria), a semiconductor diode laser with a wavelength of 1318 nm. A continuous wave (CW) was used in all cases. For laparoscopic purposes, the flexible laser fiber with the bundled light was set into a laparoscopic instrument.

### Surgical technique

**Fig. 1:**
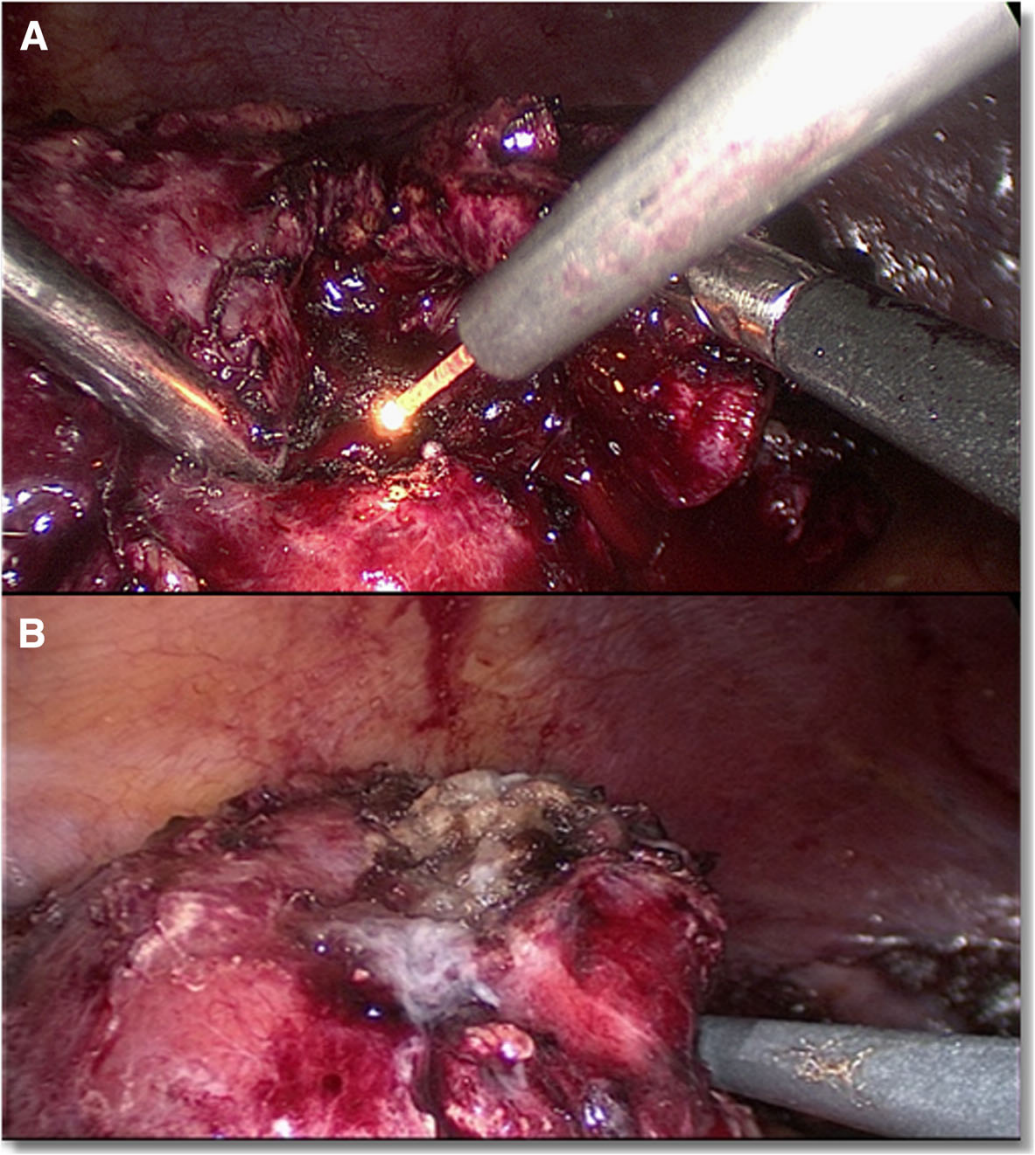
Surgical steps. **a**, Laser enucleation of the tumor. **b**, The tumor bed after enucleation and coagulation with the 1318-nm diode laser

The procedure was performed through a laparoscopic transperitoneal approach except for one case. All of the operations were performed by four different surgeons with different levels of surgical experience. By default, three trocars (Karl Storz, Tuttlingen, Germany) were set into the patient in the flank lateral position. They were inserted after achieving pneumoperitoneum with the Veress needle. The initial trocar was placed near the umbilicus with the camera port. The cranial trocar was set midway between the xiphoid and umbilicus, and the caudal trocar was placed at the edge of the rectus abdominus. In case of necessity, further trocars were put into place. After complete dissection of the kidney, we exposed the tumor outline with preservation of its overlying perinephric fat. If necessary a laparoscopic ultrasound was in standby. The surrounding fat was removed from the renal capsule. We than prepared the hilar vessels completely from the surrounding tissue, and a vessel-loop without pull was enlaced. Even while the enucleation occurred, the vessel loop was not closed. The laser fiber was introduced with a guidance instrument through a trocar. The laser power was adjusted to 45–65 W. We started applying the laser 5 mm beyond the visible tumor margin. For cutting, the laser fiber was applied in direct contact with the parenchymal tissue. For coagulation, the laser was applied 5 mm away from the tissue in a non-contact fashion and the velocity of the laser fiber was reduced (Fig. 1). Coagulation of the tumor feeding vessels before cutting was always attempted using the laser. With a dissecting forceps the tumor was mobilized in order to cut and coagulate around the tumor similar to the enucleation of an exophytic renal mass with WIT in the normal manner. Attention was paid to an adequate resection margin. During enucleation, we opened one trocar in order to preserve a good view, as there was excessive smoke. Simultaneously, suction without opening a trocar was inadequate, as the view of the operative field was diminished because of dense smoke. After enucleation, the tumor was removed by a laparoscopic sac and the resection side of the kidney inspected for bleeding (Fig. 1) before the Gerota fascia was put back into place and the closure performed in the typical fashion. The coagulation property of the laser was adequate leaving renorrhaphy omitted except for one case. All specimens were fixed in formalin and histologically examined by our Department of Pathology. Drainage was not routinely inserted. The postoperative treatment was in line with our standard operating procedures.

## Results

All patients in this study underwent tumor enucleation and coagulation with the laser only. The mean age of the patients was 67.6 (range 40–85) years, with a mean body mass index (BMI) of 27.7 (range 21.3–37.2). The mean tumor diameter was 19.4 (range 11–40) mm. Eleven of the tumors were located on the left side and 18 on the right side; 9 were in the upper pole, 6 in the midparenchymal area, and 14 in the lower pole. The Padua Score was low (6–7) in 27 patients and moderate (8–9) 2 patients. The renal nephrometry score was low (4–6) in 26 patients and moderate (7–9) in three patients. (Table 1).

**Table 1:** Characteristics of the patients and tumor size

Characteristic Median (range)	
Age, years	67.6 (40–85)
BMI	27.7 (21–37)
R.E.N.A.L. Score	4.8 (4–9)
Low (4–6): *n* = 26	
Moderate (7–9): *n* = 3	
P.A.D.U.A. Score	6.3 (6–8)
Low (6–7): *n* = 27	
Moderate (8–9): n = 2	
Tumor diameter, mm	19.4 (11–40)

The mean total operative time was 179.4 (range 90–300) minutes. The renal vessels were not clamped, except for one case in which hilar clamping had to be performed for 12 min and parenchymal sutures had to be placed because of insufficient coagulation. In all other cases, we had no WIT and parenchymal sutures were not necessary. No blood transfusions were needed perioperatively or postoperatively. The mean change in hemoglobin (Hb) from before surgery to discharge was 13.7 g/dl to 12.3 g/dl respectively, *p*-Value = 0.0001. No significant change in eGFR was measured at the 6-month follow-up; the mean decrease from before surgery to 6-month follow-up was 63,9 ml/min to 61,3 ml/min respectively p-Value = 0,27. We encountered excessive smoke when the laser was applied continuously for more than 1 min.

**Fig. 2:**
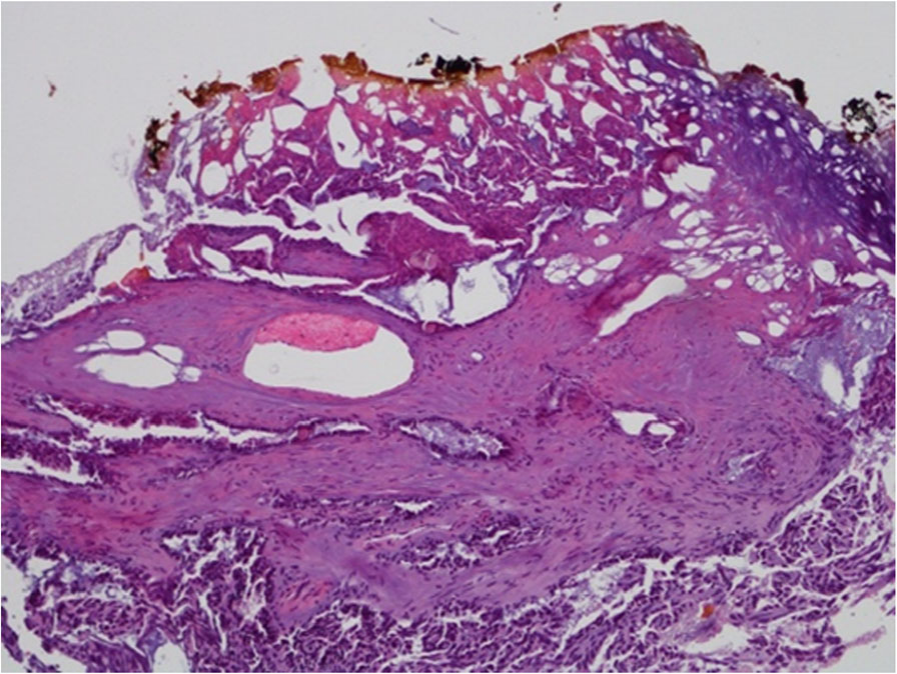
Hematoxylin & eosin staining of papillary RCC, Type 1. Magnification 100x. Note the upper part of the coagulation zone without accessibility and an adjacent fibrous pseudo capsule with tumor proliferation

Intraoperatively, no immediate frozen sections were sent to pathology and the tumor bed was evaluated macroscopically. Histologically, the majority of lesions (22/29) were renal cell carcinoma stage pT1a. Four cases of angiomyolipoma and three of oncocytoma were found. The majority of malignant lesions (13/22) were margin negative (R0). However, the interpretation of the surgical margins included 7 cases with unknown surgical margin (RX) due to charring of the tumor base through the Laser. In two cases, were classified margin positive (R1) by the pathologist (Fig. 2). As the positive margins in these two cases were described as small, they were placed into strict follow-up and a second resection avoided (Table 2).

**Table 2:** Clinical results, histopathology, and pathological classification

Clinical results	
Mean operative time	179.3 min
Mean change Hb	1.4 g/dl
Without WIT	28 Pat.
WIT	1 Pat. (12 min)
Blood transfusion	0
eGFR after 6 months	2.6 ml/min
Mean follow-up	1.8 years
Histopathology	
Renal cell carcinoma	22
Angiomyolipoma	4
Oncocytoma	3
Residual tumor classification	
R0	13 (59%)
RX	7 (32%)
R1	2 (9%)

*WIT* warm ischemic time

The mean hospital stay was 6.4 days, which is in line with the average duration of LPN in our department. No postoperative complications occurred. A mean follow-up of 1.8 years revealed no tumor recurrence.

## Discussion

Laparoscopic surgery with off-clamping of the hilar vessels and enucleation with WIT is the standard procedure for enucleation of renal masses and SRM. As LPN is a demanding operation, the WIT is still longer than with the open approach. Especially in pre-damaged organs or in the case of a single kidney, the WIT is of issue [9]. Recently the robotic partial nephrectomy has emerged as an alternative to laparoscopic partial nephrectomy. The risk of renal damage is reduced as ischaemic time is significantly shorter when using robotic surgery compared to laparoscopic surgery [13]. As the reduction in warm ischemia seems to be the best modifiable risk factor for later renal insufficiency, we wanted to investigate the feasibility of a diode laser. Reduction or omission of WIT in laparoscopic surgery of the kidney is a future goal.

The laser is a widely used tool that has been well studied in various fields of medicine but still experimental in kidney surgery. As the effectiveness of the laser is dependent on the wavelength and portion of water in the tissue, its usefulness has to be investigated. The feasibility of using a laser in the kidney was previously shown. [11, 14–28] The property of the diode laser we used (1318-nm Eraser Rolle and Rolle) was a shallow penetration depth, which leads to strong carbonization on the surface without penetrating and damaging deeper structures as it is the risk with other laser systems, such as the Ho: YAG-laser. This could lead to accidental opening of the tumor capsule, damaging deeper renal tissue. Co2 lasers, which have been tested in the past, have even less penetration depth, which leads to insufficient coagulation of larger vessels and even stronger carbonization of the tissue. For this reason, the diode laser seemed to strike a balance between these laser systems, as Khoder et al. already published their promising results with the same laser system.

To the best of our knowledge, this is the largest series of laparoscopic laser-assisted partial nephrectomy published to date. The number of patients in the literature treated with laser without WIT is even smaller, and the variety of different wavelengths used is large (Table 1).

Still the Laser is experimental when it comes to renal surgery. One major drawback during the operation was the excessive smoke building due to the carbonization of the tissue. We tried to avoid this by rinsing, which did not avoid the smoke building and the visibility was reduced. The best visibility was achieved by opening one of the trocars as a fume hood. Still, this was not optimal because the intraabdominal pressure of the pneumoperitoneum is reduced, but suction alone did not achieve good visibility. Furthermore because of the strong carbonization the surgeon was hindered to discriminate between renal tissue and tissue of the RCC.

All tumors except one were enucleated without WIT, which is great progress in terms of reducing renal damage. The main drawback in our study was the number of unclassified resection margins and positive resection margins in the histopathological examination. To the best of our knowledge, this problem has not yet been published for renal tumor enucleation. Although our pathology department is familiar with resection margins from laser enucleation in the prostate and other organs, they could not make a definitive diagnosis in 32% of the renal samples and stated them as RX. The reason for the indistinct pathological report is the carbonization of the resection margin. In addition, we had 9% positive margins, whereas 0–4% positive margins after LPN is reported in the literature. [29] The small number of patients in our cohort does not allow the general assumption of a higher rate of Rx and R1 resections. One explanation of the positive margin is the excessive smoke building during laparoscopy, which leads to difficulties in identifying the capsule. Another reason is the small diameter of the renal tumor, which has been identified as a reason for a positive surgical margin in the past. [30]

All patients with an indistinct or positive surgical margin were followed up and have had no tumor recurrence to date.

## Conclusion

Data on laser-assisted partial nephrectomy without WIT has been published for over a decade, [26] but so far this technical option is still regarded as experimental. In this retrospective study including the largest number of patients thus far, we show that the major problems are smoke building and indistinct surgical margins. In this context, we cannot promote the laser as a valid option for partial nephrectomy outside of clinical trials. As we have different results than previous clinical studies, further clinical trials are needed.

## Abbreviations

BMI: Body mass index
CW: Continuous wave
eGFR: Estimated glomerular filtration rate
Hb: Hemoglobin
LAPN: Laparoscopic partial nephrectomy
LLPN: Laser laparoscopic partial nephrectomy
LPN: Laparoscopic partial nephrectomy
MIPN: Minimal invasive partial nephrectomy
NSS: Nephron sparing surgery
PN: Partial nephrectomy
RAPN: Robot-assisted partial nephrectomy
rn: Between radical nephrectomy
SRM: Small renal masses
WI: Warm ischemia
WIT: Warm ischemic time
